# 11.E. Workshop: Urban Green Spaces, Built Environment and Urban - Mental - Environmental Health outcomes

**DOI:** 10.1093/eurpub/ckac129.704

**Published:** 2022-10-25

**Authors:** Organised by: EUPHA-URB, EUPHA-PMH, EUPHA-ENV, Chair persons: Stefano Capolongo (EUPHA-URB), Marija Jevtic (EUPHA-ENV)

## Abstract

Environmental sustainability, especially in an era of growth health inequality, is one of the most important challenges facing Public Health systems around the World. Environmental sustainability is responsibly interacting with the planet to maintain natural resources and not jeopardize the ability for future generations to meet their needs. The SDGs put environmental sustainability at the center of sustainable development. Environmental Health is the branch of Public Health concerning all aspects of the natural and built environment affecting human health. It is targeted towards preventing disease and creating health-supportive environments. It encompasses the assessment and control of those environmental factors that can potentially affect health, such as pollution, poverty and inadequate energy solutions. Urban Health is an intersectoral arena that links both the public health and the urban planning sectors, mainly captured by SDG3 (including Mental health) and SDG11. Both during the first waves of the Covid-19 pandemic period and in contemporary cities, urban environments were stressed; the resilience of our cities were tested, highlighting the strengths and weaknesses of the urban contexts, not always capable to pro-mote and protect the population health status. Urban Green Spaces (UGS) have proved essential role as “tools” to improve Urban Public and Mental Health. Unfortunately, the heterogeneous distribution of UGS inside the contemporary cities, together with the disparity in quality of such spaces, led to some exclusion phenomena. Evidence/experience-based research strongly demonstrated the positive effects on Public Health of the UGS, and for this reason, they are now becoming the strategic and challenging issue of many urban regeneration programs. The importance of UGS as a key infrastructure has generated the necessity of developing new health-centered design criteria able to conform to their new role in urban environments. The augmentation of UGS surface alone, does not necessarily make cities more livable. An increase in area and surfaces does not translate in ease of accessibility from all social groups or from all the cities’ neighborhoods, or not does it give data on the qualities of such areas, like potential for social engagement or Physical Activity.

Aim of the Workshop - organized by the three EUPHA Section URB+MEN+ENV - it would like to be to build the capacity and knowledge between participants about the main topics and urban features capable to have relevant Urban Public, Mental and Environmental Health outcomes. Additional scope is to collected case studies and research experiences considered virtuous at the international level, analyzed in detail to highlight the main urban and architectural features of those healthy experiences and the related health outcomes, such as sedentary lifestyle reduction, increase of the attractiveness of places, reduction of air and noise pollution.

**Key messages:**

• Promotion of healthy places - with particular reference to the green spaces - that enhance the experience and Mental Health needs to be part of green and inclusive recovery at all levels.

• Policy Makers, Public Health experts, civil societies & citizens are driving forces to implement the development equation, allowing cities to become greener, inclusive, safer, resilient & sustainable.

